# Genome Organization and Gene Expression Shape the Transposable Element Distribution in the Drosophila melanogaster Euchromatin

**DOI:** 10.1371/journal.pgen.0030210

**Published:** 2007-11-30

**Authors:** Pierre Fontanillas, Daniel L Hartl, Max Reuter

**Affiliations:** 1 Department of Organismic and Evolutionary Biology, Harvard University, Cambridge, Massachusetts, United States of America; 2 The Galton Laboratory, Department of Biology, University College London, London, United Kingdom; Fred Hutchinson Cancer Research Center, United States of America

## Abstract

The distribution of transposable elements (TEs) in a genome reflects a balance between insertion rate and selection against new insertions. Understanding the distribution of TEs therefore provides insights into the forces shaping the organization of genomes. Past research has shown that TEs tend to accumulate in genomic regions with low gene density and low recombination rate. However, little is known about the factors modulating insertion rates across the genome and their evolutionary significance. One candidate factor is gene expression, which has been suggested to increase local insertion rate by rendering DNA more accessible. We test this hypothesis by comparing the TE density around germline- and soma-expressed genes in the euchromatin of Drosophila melanogaster. Because only insertions that occur in the germline are transmitted to the next generation, we predicted a higher density of TEs around germline-expressed genes than soma-expressed genes. We show that the rate of TE insertions is greater near germline- than soma-expressed genes. However, this effect is partly offset by stronger selection for genome compactness (against excess noncoding DNA) on germline-expressed genes. We also demonstrate that the local genome organization in clusters of coexpressed genes plays a fundamental role in the genomic distribution of TEs. Our analysis shows that—in addition to recombination rate—the distribution of TEs is shaped by the interaction of gene expression and genome organization. The important role of selection for compactness sheds a new light on the role of TEs in genome evolution. Instead of making genomes grow passively, TEs are controlled by the forces shaping genome compactness, most likely linked to the efficiency of gene expression or its complexity and possibly their interaction with mechanisms of TE silencing.

## Author Summary

Transposable elements (TEs) are parasitic DNA segments that can move within a host genome. These selfish mobile elements are present in virtually all eukaryote species and can contribute significantly to their DNA. TEs multiply by copying themselves within the genome. Depending on where they land, new copies can alter the organism's phenotype, often negatively but sometimes positively. Although TEs have some preferences, they have few opportunities to choose their landing places. It has been proposed that new copies arise in places that are easily accessible to their insertion. Increased accessibility can occur close to genes that are actively transcribed, because the DNA is uncoiled and laid bare. We have tested whether this effect has a detectable influence on the distribution of TEs in the genome of the fruitfly, D. melanogaster. Our analysis shows that this is indeed the case. Thus, TE insertions are denser around genes expressed in the cells that give rise to sperm and eggs (the germline). This is expected because only those new copies arising in these cells are transmitted to future generations. In addition, we found that genomic regions vary in their tolerance to insertions. Thus, TEs are rare wherever a considerable increase in noncoding DNA is deleterious.

## Introduction

Transposable elements (TEs) are selfish genomic elements on the order of one to several kilobases in length. They spread by replication and insertion across the host's genome, either with the help of enzymes they encode or by parasitizing the transposition machinery provided by other elements. TEs occur in virtually all sexually reproducing species and can contribute significantly to genome size. While TEs account for only about 3% of the yeast genome, their share of the genome is roughly one half in humans and 80% in frogs [1]. Besides their abundance in genomes, TEs are of biological importance because they can affect gene and chromosome evolution in numerous ways, including insertional mutation and retrotransposition, as well as gene duplication and chromosomal rearrangements. Further, TEs have been shown to be involved in the evolution of gene expression [2–4].

The density of TEs not only differs between species, it is also very heterogeneous within genomes. Studies on the distribution of TEs in the genomes of D. melanogaster, Caenorhabditis elegans, Arabidopsis thaliana, and humans have shown that elements tend to be enriched in regions of low gene density [5–8], with the notable exception of human SINE elements [7]. In D. melanogaster, TEs account for 50%–60% of the heterochromatic regions and for only 4%–6% of the euchromatin [5,9]. Moreover, only 28% of euchromatic TEs occur within genes, although genes make up over half of the euchromatic DNA [10,11]. Similar to gene density, local recombination rate has been shown to correlate negatively with TE density ([12], but see [6,13]). Again in D. melanogaster, TE density is more than 6-fold higher in genomic regions with little or no recombination than those with high recombination [14].

The genomic distribution of TEs has been interpreted as the result of selection against the deleterious effects of insertions. Negative selection is thought to result either from the insertion of TEs into functional regions or from ectopic recombination, events of crossing-over between identical elements at different chromosomal positions, which generate deleterious chromosome rearrangements [15]. Under both mechanisms TE density is predicted to increase with low recombination rate, either because Hill-Robertson interference reduces the efficiency of selection against deleterious insertions or because the probability of ectopic recombination declines [16].

This general picture appears to suggest that the distribution of TEs is determined primarily by the removal of new insertions because of the action of selection, while variations in insertion rate across the genome contribute comparatively little to the distribution of TEs. This impression might be biased by the fact that studies assessing the variation in TE density have been performed at a very large genomic scale, thus putting factors potentially modulating insertion rate beyond their scope. Studies of insertion rates, in contrast, have mostly been restricted to identifying the features of insertion sites of a few specific TE families [17–20]. Because of this dichotomy in approaches, little—if anything—is known about general factors modulating the insertion rates and their relative contribution in generating variation in the local density of TE insertions across genomes.

One candidate factor able to modulate insertion rates is gene expression. Bownes [21] was the first to propose the idea that transcriptional activity could favor insertion, based on the empirical observation of *P* element insertion patterns in fruitflies. Insertion bias towards expressed genes can be explained mechanistically: transcription is associated with a decondensation of the chromatin, which renders the DNA accessible to the transcriptional machinery but potentially also to the enzymes involved in transposition [22,23]. The effect of gene expression on insertion rate can be assessed relatively easily, because it will lead—over successive generations—to an accumulation of element insertions in and around germline-expressed genes relative to soma-expressed genes. This differential accumulation arises from the fact that only those transposition events taking place in the germline are transmitted to future generations, whereas all somatic insertions are lost. So far, differential accumulation has only been studied in the *P element* and over the short term (over one generation), by identifying new insertions after the artificial mobilization of elements [21,23]. While these studies indicate the existence of an expression-related insertion bias, they cannot inform us about the generality of such a bias or its relative importance compared to forces of counterselection. Addressing this question requires an analysis at a genomic scale that is able to detect the effects of both insertion bias and counterselection for all element types and over many generations.

In this paper, we present such an analysis of the fine-scale distribution of TEs in the D. melanogaster euchromatic genome. We focus on the question of whether gene expression favors TE insertion but also take into account other parameters of genome organization that have been shown or can be expected to influence TE distribution. Our results show that insertion bias towards germline-expressed genes has a detectable effect on the distribution of TEs in the D. melanogaster genome. However, the effect is confounded and overridden by the fact that germline-expressed genes are under strong selection for compactness (against excess noncoding DNA), compared to soma-expressed genes. We show that, along with recombination rate, selection for local genome compactness is the major determinant of local TE density in the fruitfly. Furthermore, both of these factors are related to the organization of the genome into coexpressed gene clusters. As a consequence, the fine-scale distribution of TEs is strongly shaped by genome architecture.

## Results/Discussion

### Factors Affecting TE Distribution

We analyzed the distribution of 5,062 TE insertions annotated in the genome sequence of the D. melanogaster reference strain [24] (see Materials and Methods for details). The genome sequence contains annotated insertions for a total of 151 TE families including the interspersed element 1 (INE-1), which accounts for ∼40% of euchromatic insertions. No other TE family exceeds 5% of the total number of insertions, but two-thirds of the families are represented by at least five copies (Tables S1–S3). We located all TEs based on the annotations and classified them as mapping to UTRs, exons, introns, or intergenic regions. Three expressed sequence tag (EST) libraries (head, testis, and ovary) allowed us to classify 1,829 genes as exclusively expressed in somatic tissue (head) and 2,388 genes as exclusively expressed in germline cells (testes or ovaries). These two classes of genes (exclusively germline- or soma-expressed) are expected to show contrasted effects of gene expression on TE distribution and hence to maximize the statistical power of our analysis. We have, in addition, performed an alternative analysis that does not rely on a strict classification of genes. Instead, this approach takes advantage of a recently published Affymetrix microarray dataset (FlyAtlas, http://www.flyatlas.org/, [25]) that contains the expression levels of 13,046 genes measured in male and female germline as well as eight somatic tissues. The results of this alternative approach are in complete agreement with conclusions of the EST approach (see Text S1).

We used the statistical framework of Generalized Linear Models (GLMs) to analyze the distribution of TEs in the genome. Specifically, we modeled the number of TEs mapping to genes (transcripts: exons + introns + UTRs) or intergenic regions as a function of several parameters describing the properties of these genomic entities. Two parameters captured aspects of gene expression. One designated the expression of the element itself as germline- or soma-expressed, whereby intergenic regions were classified as “germline-expressed” if at least one of the adjacent genes was expressed in the germline. A second variable captured the broader expression context as the proportion of germline-expressed genes among the ten closest neighbors of a gene/intergenic region. This parameter allowed us to assess whether germline-expression can affect TE insertion in more distant genes. The window size of 10 was chosen on the basis of pilot analyses assessing the effect of germline expression among 20 neighbors on TE number in a focal gene/intergenic region (Figure S1).

In addition to the variables describing gene expression, we entered four measures of genomic context. The first was recombination rate, which has been shown to have a profound impact on TE distribution [12,14,26]. Recombination rates are not distributed randomly with respect to gene expression; they are greater around soma-expressed genes than germline-expressed genes (medians: 2.75 versus 2.58 cM/Mb; Wilcoxon rank test, *p* < 0.01). The second genomic feature used was the amount of noncoding DNA, excluding TE lengths. This is of importance because virtually all TE insertions in the D. melanogaster genome reside in noncoding DNA [10], and because noncoding length is also correlated with gene expression. Indeed, germline-expressed genes have shorter noncoding sequences (median 567 bp, introns + UTRs) than soma-expressed genes (1,212 bp, Wilcoxon rank test, *p* < 0.001), and germline intergenic regions are shorter than soma intergenic regions (577 bp versus 813 bp, *p* < 0.001). Third, we included the proportion of noncoding sequence that is evolutionarily conserved [sensu 27]. Sequence conservation is important because it is thought to be associated with a functionality [28–30]. Since insertions in functional elements are likely to be deleterious, the degree of sequence conservation reflects the portion of the noncoding sequence in which new insertions will be rapidly eliminated by selection. Furthermore, sequence conservation is a highly relevant covariable in our particular analysis because it is also correlated with gene expression; the proportion of conserved sequences is smaller in germline-expressed as compared to soma-expressed genes (12.9% and 14.7%; Wilcoxon rank test, *p* < 0.001) and smaller in germline intergenic regions than in soma intergenic regions (12.5% and 13.1%, respectively; *p* < 0.001). Finally, our statistical model includes a factor describing the chromosomal position of a gene or intergenic region as X-linked or autosomal. This distinction is important because male hemizygosity for the X chromosome entails a difference in the intensity of selection between the X and autosomes [16,31].

The GLM was able to account for more than 35% of the variance in TE number between genes and intergenic regions (Table 1). The analysis showed that TE number is affected both by genomic features and by gene expression. We will first briefly summarize the results before discussing the main new points in more detail. As indicated by previous studies, local recombination rate has a negative effect on TE density. In our GLM analysis, the effect of recombination rate accounts for almost a fifth of the variance in TE number between genes/intergenic regions, and it is highly significant. However, the analysis also revealed important effects of genomic context that had not previously been described. Notably, noncoding length—a measure of genome compactness—has a highly significant and positive effect on TE density, indicating that TEs accumulate in regions of the genome that are less compact. This factor explains a portion of the variance that is comparable to that accounted for by recombination rate. The remaining two genomic features, proportion of sequence conservation and chromosomal location, also have significant effects but each explains only a small part of the variance in TE number. Finally, gene expression affects TE density in a significant way. However, as indicated by several significant interaction terms, the effect of gene expression is dependent on genomic context.

**Table 1:**
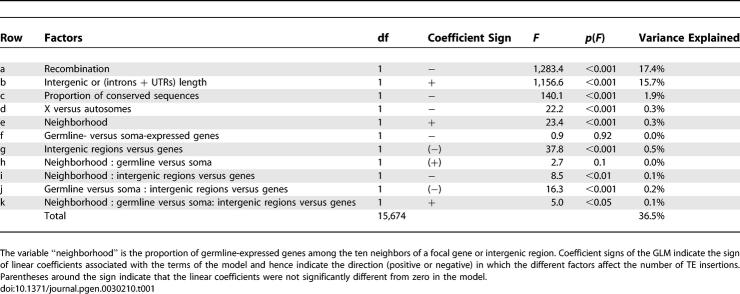
Factors Affecting the Number of TE Insertions in Genes and Intergenic Regions (GLM Analysis)

### Sequence Conservation

As shown in Table 1 (row c), the degree of sequence conservation has a significant effect on TE density (*F_1,15663_* = 139.5, *p* < 0.001) and explains 1.9% of the total variance in TE number between genes/intergenic regions. The factor has a significant negative coefficient, indicating that the number of insertions decreases with the portion of noncoding sequence that is conserved between *Drosophila* species. This result is interesting because sequence conservation is an indication of purifying selection on the sequence and hence reflects functionality. In the case of the noncoding sequences considered here, conservation most likely arises because part of the sequence is composed of regulatory elements. Our result therefore indicates that, as expected, insertions of TEs into regulatory elements causes a deleterious fitness effect, just as insertions into coding sequences do.

### Genome Compactness

**Figure 1:**
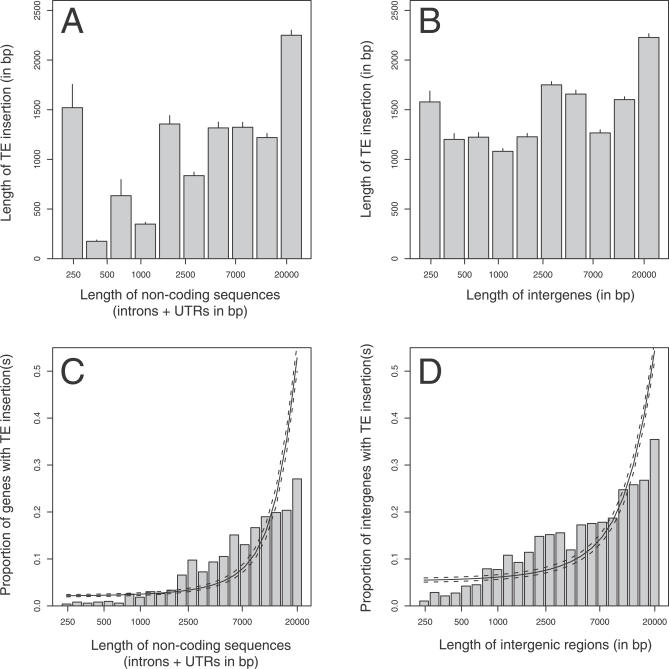
TE Insertions and Selection for Genome Compactness (A,B) The correlation between the length of noncoding sequences and the length of TE insertions for genes and intergenic regions. To avoid interaction or facilitation effects in the case of multiple insertions, only unique insertions (by gene or intergenic region) were incorporated in this analysis. (C,D) The nonlinear relationship between the length of noncoding sequences and the proportion of genes or intergenic regions with TE insertion(s). The lines represent the linear model (estimate and standard error) for this correlation (quasibinomial GLM on presence/absence of insertion[s] in genes and intergenic regions).

Our GLM analysis shows that TE distribution is shaped to a large degree by local variations in genome compactness. For both genes and intergenic regions, the number of insertions increases with the length of noncoding sequences (Table 1, row b). This correlation is not unexpected. Indeed, if TEs insert randomly and insertions are selectively neutral, long intergenic regions and genes with more noncoding DNA (introns + UTRs) should have more TEs because they represent a larger target for insertion. However, several pieces of evidence suggest that insertions in noncoding sequences are not selectively neutral and that they are not only affected by the presence of regulatory elements but also by local selection for genome compactness. First, according to the GLM analysis, intergenic regions accumulate about 75% more TEs than introns and UTRs. A difference in TE density between genes and intergenic regions had been found earlier [11]. However, by correcting for both noncoding length and sequence conservation, our analysis demonstrates that a difference in TE density exists even between noncoding DNA within and outside genes. Such a difference would not be expected if target size alone determined the number of TE insertions. Second, the canonical length of TEs (the length of the functional element at the moment of insertion) tends to be positively correlated with the noncoding length of the genes (Spearman rank correlation, rho = 0.11, *p* = 0.075) and intergenic regions (rho = 0.24, *p* < 0.001) that they are inserted in. Thus, long functional TEs are less likely to be retained in short intergenic regions and short genes than in less compact intergenic regions and genes. In addition to this difference in retention, compact genes also seem to eliminate their TE insertions quickly. Accordingly, the amount of TE degradation (the difference between canonical and present length) is negatively correlated with the noncoding length of genes they are inserted in (rho= −0.21, *p* = 0.001; intergenic regions rho= −0.11, *p* = 0.15). Because of the combination of these two effects, we observe a significant positive relationship between the present length of TEs and the noncoding length of the genomic entities they are inserted in (genes: rho = 0.22, *p* < 0.001; intergenic regions: rho = 0.11, *p* < 0.01; Figure 1A and 1B).

Assuming that insertion rates are independent of the length of both the TE and the targeted intron or intergenic region, the above data suggest that short intergenic regions and in particular short genes are under selection for compactness. Accordingly, TE insertions elongating these compact regions are deleterious and will be eliminated either immediately or degraded by deletions more quickly. Long genes and intergenic regions, on the other hand, seem to be more tolerant to insertions. This relationship between compactness and selection against insertions could explain the nonlinear correlation between the proportion of genes and intergenic regions containing TE insertions and the lengths of noncoding sequences (Figure 1C and 1D). On the other hand, a nonlinear relationship could be also generated by an insertional rate depending on the size of the target. Regardless of the relative contribution of these two effects, selection for compactness or insertional rates, the nonlinear relationship has an important implication: TE insertions are restricted to a limited number of genes with very large noncoding content, whereas genes with short noncoding sequences are virtually free of TEs (Figure 1C). Thus, 99% of the insertions are concentrated in the 61% of genes in which the noncoding length is greater than 500 bp.

Selection for genome compactness could arise for a variety of reasons. Within genes, compactness has been suggested to be selectively advantageous because it reduces the cost of transcription [32,33]. It has also been shown both in vitro and in vivo that long introns increase the rate of exon-skipping, the erroneous splicing of an exon [34]. From this perspective, the elongation of an intron will cause a greater increase in deleterious alternative splicing in genes that use to have short introns compared to less compact genes. Why compactness per se should be selected for in intergenic regions is less clear. Recent work has provided evidence for the existence of groups of coexpressed genes in eukaryotes, similar to bacterial operons [35]. In this context, short intergenic regions might be selectively advantageous in that they facilitate the coordinated expression of adjacent genes.

### X-Linkage

According to our GLM analysis (Table 1, row d), the density of TEs is significantly higher on the X chromosome than on autosomes. A higher density of TEs on the X chromosome could be expected for two reasons. First, selection against TEs due to deleterious effects of insertions can be less efficient on the X, since theoretically the X chromosome has a lower effective population size than autosomes. Whether this is actually the case is currently unclear. Based solely on the fact of male hemizygosity for X-linked genes one would predict a reduced effective population size for X-linked genes compared to autosomal genes [31]. However, this difference can be reduced or even reversed if males vary more in their reproductive success than females [36]. Genetic data from D. melanogaster are inconsistent, showing that genetic polymorphism is higher on the X compared to autosomes in African populations, whereas the contrary is true for populations sampled outside Africa [37–39]. Thus, it is difficult to judge whether an increased TE density on the X could be the result of inefficient selection against deleterious insertions.

Alternatively or in addition to increased drift, the increased density of TE insertions on the X could be explained by an effect of dosage compensation. In *Drosophila*, male hemizygosity for the X chromosome is compensated by doubling expression of all X-linked genes, which adjusts mRNA levels in males to those of females bearing two X chromosomes. This effect has been shown to be associated with an alteration of the chromatin structure that spreads along the chromosome [40]. Given this large-scale change in the accessibility of X-chromosomal DNA, dosage compensation has the potential to increase TE insertion rate along the entire X chromosome, in a manner equivalent to the localized effect of gene expression. Consistent with this hypothesis, Pasyokova and Nuzhdin [41] found that new copia insertions occur more frequently than expected on the X chromosome. However, the same is not true for another element family (Doc)*.*

### Germline Gene Expression

**Figure 2:**
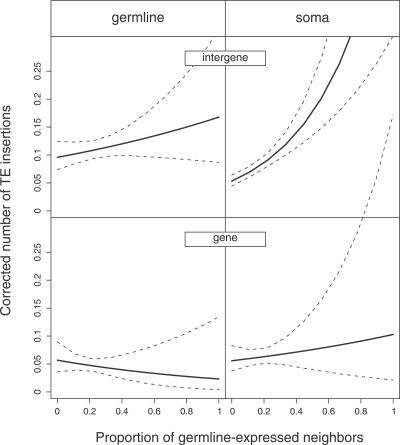
Number of TE Insertions in Intergenic Regions and Genes as a Function of the Proportion of Germline-Expressed Neighbors The figure represents the coefficients (±95% confidence intervals) from the GLM analysis presented in Table 1. The number of TE insertions per gene and intergenic region shown on the *y*-axis is corrected for the effect of four covariables entered in the GLM (cf., Table 1): recombination rate, intergenic region or intron + UTR length, proportion of conserved elements, and chromosome (X versus autosomes).

The GLM analysis supports the hypothesis that gene expression increases the probability of TE insertions. However, the effect depends on the genomic context. Figure 2 is a graphical representation of the GLM analysis (Table 1) and shows the effects of the expression context (the proportion of germline expressed genes in the neighborhood of a focal gene) on genic or intergenic TE density. As illustrated by this figure, the effect of expression in intergenic regions is straightforward: regions adjacent to germline-expressed genes tend to have more TEs than those next to soma-expressed genes. In addition, the density of TEs increases in a highly significant manner with the proportion of neighbors that are germline expressed. Patterns of insertion are more complex for genes: while soma-expressed genes, like intergenic regions, accumulate more TEs when they are surrounded by more germline-expressed neighbors, the trend is reversed among germline-expressed genes. Here, TE number is higher when the proportion of germline-expressed neighbors is lower.

The difference in the neighborhood effect between the two types of genes can be understood as the result of a combination of two factors previously described: the differences in the compactness of genomic regions and the virtual absence of TE insertions in genes with noncoding content < 500 bp. As also already mentioned, the genome of D. melanogaster is very compact around germline-expressed genes. Their noncoding sequences (introns and UTRs) are less than half as long as those in soma-expressed genes. In other words, only 53% of germline-expressed genes have a length of noncoding sequence > 500 bp, whereas the value is about 71% in soma-expressed genes. This difference in compactness translates immediately into the frequency of TE insertions in the two types of genes: while TE insertions are present in only 4.2% of germline-expressed genes (100 out of 2,388), they occur in 8.7% of soma-expressed genes (159 out of 1,829). This difference is highly significant (Chi^2^= 205.7, *p* < 0.001). On the other hand, although flanking intergenic regions are 30% shorter around germline-expressed genes than around soma-expressed genes, the prevalence of TE insertions is not significantly different (Chi^2^= 0.9, *p* = 0.33): insertions occur in 10.2% and 10.9% of germline and soma intergenic regions, respectively (see Table S2).

**Figure 3:**
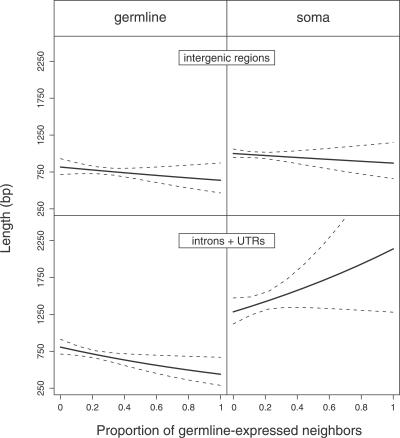
Length of Intergenic Regions and Introns + UTRs as a Function of the Proportion of Germline-Expressed Neighbors The figure represents the coefficients (±95% confidence intervals) of a log-gaussian GLM analysis of noncoding length of genes/length of intergenic regions as a function of the factors *tissue of expression* (germline versus soma), *element* (gene versus intergenic), and *neighborhood* (proportion of germline-expressed genes among the ten neighbors). The figure illustrates that the effect of the genomic neighborhood depends both on the identity of the genomic element (gene versus intergenic region) as well as the expression type, a fact reflected in the significant triple interaction term in the GLM (*F* = 4.19, *p* < 0.05).

Genome compactness around a gene not only depends on its tissue of expression but also on the gene expression of its neighbors. As shown in Figure 3, in germline-expressed genes, compactness increases even further if the neighboring genes are also germline expressed. On average, 53% of germline-expressed genes have a length of noncoding sequence > 500 bp. However, this value climbs as high as 58% in a germline-free environment whereas it drops to 38% in an environment containing 50% of germline-expressed neighbors. Consequently, stretches of germline-expressed genes have a low probability of accumulating TEs since the average length of their noncoding sequences is below the threshold of TE insertion. Soma-expressed genes, in contrast, are not only generally less compact but also increase in noncoding content when they are surrounded by more germline-expressed genes.

Our additional analyses based on germline-expression level suggest that the expression in ovaries affects the distribution of TEs more strongly than the expression in testes (see Text S1). Thus, ovary-expression level has a stronger effect on TE density in neighboring genes than testis-expression level. Furthermore, TE density is also higher within genes with higher levels of ovary-expression, although it is not clear whether this effect is due to their expression or due to the fact that ovary-expressed genes are less compact than testis-expressed genes.

### Genome Organization and Transcriptional Territories

Figures 2 and 3 show how the distribution of germline- and soma-expressed genes within the D. melanogaster genome affects genome compactness, and hence the TE density. For this analysis, we assessed the genome organization in terms of tissue of expression (more precisely, the proportion of germline-expressed genes among ten neighbors). However, other studies have evaluated genome organization in terms of level of gene expression, i.e., the amount of mRNA. In a wide range of eukaryotes, these studies have shown that coexpressed genes are not randomly distributed in the genome but are clustered into contiguous regions [42–46]. These transcriptional territories are often interpreted as chromosomal domains whose expression is regulated through a higher-order control of chromatin packaging [47]. In D. melanogaster, Spellman and Rubin [48] described 211 transcriptional territories containing between four and 45 genes (total: 3,325 genes; Tables S1–S4). Based on our classification by tissue of expression, these clusters are not tissue specific in their expression (Table S4) but are enriched in clusters of germline- and soma-expressed genes compared to the rest of the genome (Table 2; Figures S2 and S3). This therefore suggests that gene expression is a noisy process, as opening chromatin to express one gene might incidentally allow leaky expression of neighboring genes [49]. In addition, transcriptional territories differ in lengths of noncoding content (Figure S4), recombination rates (Figure S5), and proportion of conserved sequences (Figure S6).

**Table 2:**
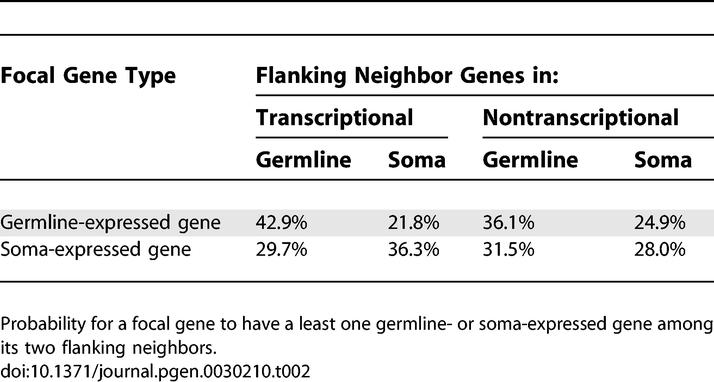
Gene Clustering in Transcriptional and Nontranscriptional Territories

Transcriptional territories are interesting for understanding the factors affecting TE density for two reasons: they represent regions of the genome that have an unusual gene clustering, and they represent regions of the genome where TE insertion rate could be modulated homogeneously over long stretches of DNA through the action of a unique mechanism of gene expression regulation. Indeed, if chromatin packaging controls the accessibility of several genes at the same time, we expect to observe a correlation between TE density and the proportion of germline-expressed genes in transcriptional territories, whereas no difference in TE accumulation should be found between soma- and germline-expressed genes within territories. To test this hypothesis, we set up a second GLM that analyzed the total number of TE insertions per transcriptional territory. The model explains a remarkable 63% of the total variance (Table 3). It confirms that TEs accumulate preferentially in transcriptional territories that have a low recombination rate, a high noncoding DNA content, a low proportion of conserved sequences, and are situated on the X chromosome (Table 3, rows a–d). Furthermore, as expected, transcriptional territories enriched in germline-expressed genes contain a higher density of TEs: the proportion of germline-expressed genes per transcriptional territory accounts for 4.5% of the total variance in the data (Table 3, row e). Importantly, this effect is specific to clusters and cannot be found to a comparable degree when analyzing random groups of adjacent genes (Table S5; see Materials and Methods).

**Table 3:**
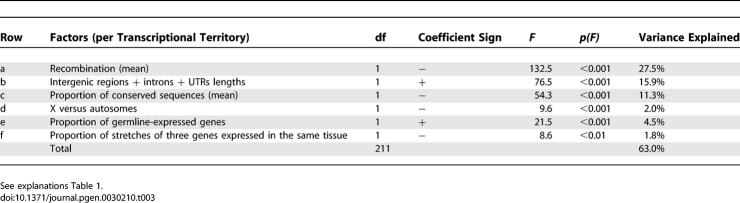
Factors Affecting the Total Number of TEs within Transcriptional Territories (GLM)

**Figure 4:**
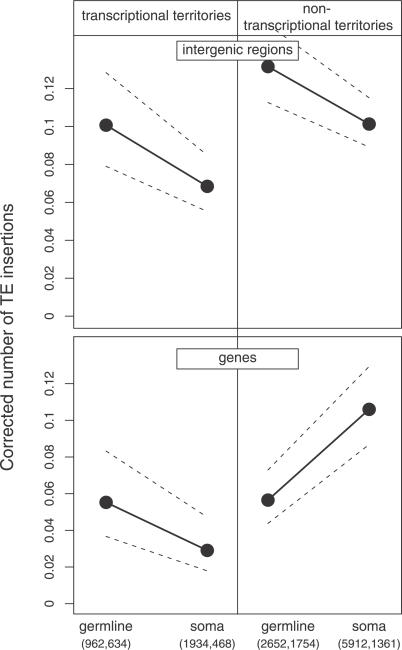
Number of TE Insertions for Genes and Intergenic Regions in Transcriptional and Nontranscriptional Territories This figure is a representation of a GLM analysis including *recombination*, *noncoding length*, *proportion of conserved sequences, chromosome* (X versus autosomes)*, territory* (transcriptional territories versus nontranscriptional territories), *tissue of expression* (germline versus soma), and *element* (gene versus intergenic regions) (see Table S6). The triple interaction among the last three factors is significant (*F* = 8.8, *p* < 0.01). The figure shows the coefficients and their 95% confidence intervals and the number of intergenic regions and genes, respectively.

To separate the effects related to genomic organization from those of gene expression, we also included in the model the probability of observing stretches of three genes expressed in the same tissue. Although this variable is not completely independent of the proportion of germline-expressed genes, the correlation is relatively low (Spearman rho = 0.35, *p* < 0.001). In the GLM (Table 3, row f), this variable is significant and explains a small part (1.8%) of the variance. Contrary to our prediction, TE density is also not homogeneous within transcriptional territories: germline-expressed genes and their intergenic regions accumulate more TEs than soma-expressed genes and their intergenic regions (Figure 4; Table S6).

In summary, these results suggest that the accessibility of DNA in transcriptional territories is affected by properties of the transcriptional territory as a whole (i.e., the proportion of germline expression). However, the factors associated with individual genes and their neighbors, which are important outside transcriptional territories, still have some effect on TE density. It therefore appears that the variations in TE insertion rates affecting a transcriptional territory as a whole are partially overridden by differential selection on individual genes.

### Conclusions

In this paper, we analyzed the fine-scale distribution of TEs in the D. melanogaster euchromatic genome. Transposition is a highly stochastic process. Accordingly, the distribution of TEs is affected by randomness in both the location of new insertion and their retention through successive generations in the face of genetic drift, as well as by the idiosyncrasies of individual element families in their modes of transposition and target site preferences. Despite the inherent randomness of the data and potential measurement error in the predictor variables, our analysis has revealed a number of highly significant effects that are strong enough to be detected in a global analysis such as the one presented here. Thus, in agreement with earlier studies [12,14,26,50], we found that recombination rate is a major determinant of TE density across the genome; regions that recombine more frequently have fewer TE insertions. In addition, our study identified factors modulating TE density whose importance had hitherto not been recognized: genome compactness (the length of noncoding DNA) and gene expression. Regarding TE distribution, genome compactness encapsulates two effects: the size of the target and the tolerance to insertions. From a probabilistic point of view, long stretches of noncoding DNA are more likely to be the target of TE insertion. However, our analysis also shows that regions of the genome also vary in their tolerance to TE insertions. First, intergenic regions are typically more tolerant to TE insertions and accumulate more TEs than introns or UTRs. Second, compact regions of the genome are less tolerant to insertions than regions with large amounts of noncoding DNA, resulting in the rapid elimination of insertions by natural selection.

Selection against new insertions into compact genes could arise for several reasons. First, selection against insertions could be due to the reduction in the efficiency of splicing caused by the elongation of introns. It has been shown that long introns are associated with increased levels of alternative splicing, both in vitro and in vivo (*Drosophila* and humans) [34]. Accordingly, the elongation of an intron through the insertion of a TE will lead to a larger increase in the level of accidental alternative splicing in compact genes with short introns than in genes in which introns were initially large. Second, insertions into compact genomic regions could be deleterious because they reduce the efficiency of gene expression. The amount of noncoding DNA within genes has been shown to decrease with both the level and the breadth (number of different tissues) of expression in a variety of species, consistent with a cost of transcribing noncoding sequence [32,33]. Following this interpretation, TE insertions into compact genes would be deleterious by increasing the cost of transcription. Third, we could also speculate that TE insertions into compact regions are counterselected because of the epigenetic silencing they induce in their vicinity. In D. melanogaster TEs can be silenced by chromatin modifications (formation of localized heterochromatin, see [51,52] for reviews). Chromatin-based silencing might be deleterious in compact genomic regions, because the heterochromatic structure can spread into the regulatory sequences of adjacent genes [53]. Fourth and finally, selection against TE insertions into compact genes could arise from the association between compactness and germline expression described above, in conjunction with germline-specific TE silencing. D. melanogaster germline cells (but not somatic cells) suppress TE activity with the help of repeat-associated short interfering RNAs (rasiRNAs), which target and degrade transcripts containing repeat sequences [54]. As a consequence of this silencing, TE insertions within germline-expressed genes are extremely deleterious. Indeed, the presence of insertions not only causes the degradation of TE transcripts but also the degradation of mRNA produced by genes bearing insertion. The strong selective pressure exerted by this post-transcriptional silencing mechanism could explain the quasi-absence of TEs within germline-expressed genes and may help to maintain the compactness of these genes.

Despite the deficit of TE insertions in germline-expressed genes, our study demonstrates that germline expression increases the local rate of TE insertion. Thus, TE insertions are denser around germline-expressed genes than elsewhere in the genome, unless these regions are under selection for increased compactness. This result is consistent with the positive effect of germline expression on insertion rate observed in experimental studies of novel *P element* insertions [21,23]. Our study shows that this effect is general, rather than specific to the *P element*, and significantly shapes the genomic distribution of TEs. Furthermore, the fact that the signal of expression-related insertion bias can be detected across coexpressed gene clusters provides evidence that higher TE accumulation is associated with chromatin decondensation, as speculated earlier [21,23]. Taken together, these results make a strong case for an expression-associated increase in TE insertion rates. It is not impossible that other factors contribute to the association between germline expression and TE density. For example, germline-specific TE silencing could reduce the deleterious effects of TEs inserted close to germline-expressed genes and thus decrease the strength of counterselection in these positions relative to selection in the vicinity of soma-expressed genes. While conceivable as a mechanism, the molecular basis of such an effect remains speculative in the absence of empirical evidence.

**Figure 5:**
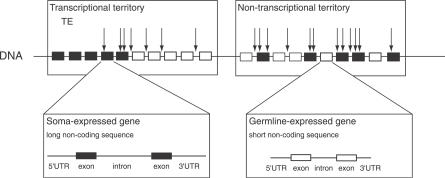
Schematic Representation of TE Dynamics in the Euchromatin of D. melanogaster This figure illustrates the distribution of TEs in and between germline-expressed and soma-expressed genes (in white and black, respectively) within and outside transcriptional territories.

The factors discussed above—recombination rate, noncoding length, gene expression, transcriptional territories, and TE silencing—can be shown to have (sometimes strong) individual effects on local TE density. However, our analysis has also made clear that all of these genomic variables interact to create a complex genomic landscape that in turn shapes TE distribution (Figure 5). Thus, germline-expressed genes are compact, with short noncoding sequences, and are even more so when organized in groups of adjacent germline-expressed genes. As a consequence of their compactness and the selective forces driving it, germline-expressed genes accumulate few TEs. Inversely, soma-expressed genes have a higher noncoding content. Accordingly, they accumulate many TEs when located among germline-expressed neighbors. Transcriptional territories thus accumulate fewer TEs than nontranscriptional territories (Figure 4) since they are enriched in gene clusters: germline-expressed genes are extremely compact, and the soma-expressed genes are not strongly exposed to the transpositional effects of germline-expressed neighbors. Because of the inter-relationships between noncoding content and tissue of expression revealed by our study, genome organization emerges as one of the major determinants of TE distribution, on a par with recombination rate.

The direct link between genome compactness and TE density identified here is not only important to understand the distribution of TEs in the D. melanogaster genome, but also sheds a completely new light on the role of TEs in genome evolution. The current view is that TEs are responsible for the growth in the noncoding part of genomes [55] and are thus a driving force in the evolution of genome size. However, our work shows that TE insertions can be deleterious just by elongating the noncoding part of otherwise compact genes, and TEs are restricted to regions of the genome where noncoding DNA is tolerated. Thus, rather than making genomes grow passively, TEs are controlled by the forces shaping genome compactness, most likely linked to the efficiency of gene expression [32,56] or its complexity [29,57]. It is possible that selection against noncoding DNA affects TEs more strongly in D. melanogaster with its compact genome [58,59] compared to species with larger genomes. However, variations in noncoding content between genes are a general phenomenon [60,61] and, as a consequence, TEs will be subject to selection against noncoding DNA across organisms.

Finally, our results on insertion bias towards germline-expressed genes add an interesting perspective to recent work on duplicate genes arising by TE-mediated retrotransposition (retrogenes). Recent studies have shown that new retrogenes are generally expressed in germlines, in particular in testis [62,63]. So far, this expression bias has been attributed either to the fact that gene expression is generally incremented in testes or to the fact that retroposition from the X to the autosomes can provide a way for genes to escape the inactivation during spermatogenesis that affects X-linked genes. The insertion bias documented in our study suggests that testis expression is to be expected for retrogenes, because they preferentially insert into or close to germline-expressed genes. Since gene expression is a leaky process [49], a new retroposed duplicate gene is then likely to be transcribed in germ cells, merely because it is surrounded by germline-expressed neighbors. We also observed a greater transposition activity in the X chromosome than in autosomes, and we could envisage more retrogenes on the X chromosome. However, this prediction seems to be in contradiction with the observed excess of functional retrogenes that originate from the X chromosome and retropose to autosomes in D. melanogaster [64]. Based on this discrepancy, it appears that the recruitment of duplicate genes, relative to their chromosomal location, is more immediately affected by natural selection rather than insertion bias. This interpretation is in agreement with data from human and mouse, suggesting that movements of genes by retroposition are affected by selection more than random processes [65].

## Materials and Methods

### Genomic data.

The 13667 annotated genes in D. melanogaster genome (release 4.2.1) were downloaded from FlyBase (ftp://ftp.flybase.net/genomes/Drosophila_melanogaster/current/fasta/). We discarded 621 overlapping genes. We extracted the lengths of intergenic regions, as well as the coding sequence (CDS) and the noncoding sequence (introns and UTRs) of each gene after excluding the TE lengths. Recombination rates were obtained from Hey and Kliman [66] (http://lifesci.rutgers.edu/~heylab/HeylabData.htm). We used the *R* estimate, which is based on a comparison of the genetic and physical map locations of 493 X-linked and autosomal genes. Conserved sequences were obtained from genome-wide multiple alignments of eight insect species (http://genome.ucsc.edu/) [27]. The 50,000 top-scoring elements were used, accounting for approximately 14% of the *Drosophila* euchromatin. The conserved sequences overlapping TEs were excluded.

### Germline and soma-expressed genes.

To classify the genes as germline- and soma-expressed, we used three published EST libraries (Berkeley Drosophila Genome Project). The AT (adult testes) library was made from RNA extracted from D. melanogaster adult male (0–3-d-old nonisogenic Oregon-R strain) testes and seminal vesicles. The AT library contains 23,505 EST from 3,921 genes present in the FlyBase release 4.2.1. The GM library was made from RNA extracted from ovaries, at stage 1–6 of oogenesis (nonisogenic Oregon-R strain). The GM library contains 11,151 EST from 3,152 genes. Finally, the RH (Riken head) library was made from RNA extracted from adult heads (isogenic *y; cn bw sp* strain). The RH library is normalized and contains 51,487 EST from 4,361 genes.

Each library includes tissue-exclusive genes and genes shared with the two other libraries. The 1,829 genes present exclusively in the RH (head) library have been called soma-expressed genes. The 1,567 and 821 genes present exclusively in the testis (AT) and ovary (GM) libraries, respectively, have been called germline-expressed genes.

### Germline and soma intergenic regions.

We categorized intergenic regions by looking at the tissue of expression of the two flanking genes: an intergenic region surrounded by one or two germline-expressed genes is called germline intergenic. Conversely, a soma intergenic region has no germline-expressed genes among its close neighbors.

### Transcriptional territories.

Spellman and Rubin [48] described 211 large groups (transcriptional territories) of adjacent genes that are similarly expressed (Table S4). Their analysis was carried out on the basis of 267 GeneChip Drosophila Genomes Arrays (Affymetrix) from 88 different experimental conditions studying diverse biological processes (aging, immune response, etc.). Consequently, these transcriptional territories are neither tissue specific, nor sex specific (see [67] for testis or ovary clusters), nor are they function specific (see [68] for dosage compensation clusters). Rather, they are believed to represent a higher order of function-independent expression regulation that takes place at the level of the chromatin structure (but see [35]). We calculated descriptive statistics for each territory: the number and the mean length of genes, the total length covered by genes, the total length of intergenic sequence, and the proportion of germline-expressed genes. Each cluster has between four and 45 genes (total: 3,325 genes) and covers between 23 and 553 kbp. They contain on average 19.7% (range: 0%–60%) and 14.0% (0%–50%) of germline- and soma-expressed genes, respectively.

### Random chromosomal territories.

We generated 500 random datasets of chromosomal territories by sampling contiguous gene clusters after excluding transcriptional territories. Each dataset has the same structure as the dataset of transcriptional territories (211 clusters containing between four and 45 genes [total: 3,325 genes]).

### TE dataset.

We downloaded all annotated natural TE insertions from FlyBase (release 4.2.1; ftp://flybase.net/genomes/Drosophila_melanogaster/current/fasta/) [24]. The complete dataset contains 6,006 insertions from 159 TE families, but only 5,060 of these insertions are within genes and intergenic regions (Table S2). One TE (interspersed element 1, INE-1) is predominant, accounting for ∼40% of insertions, and the others never exceed 5% each. The DNA transposons (class II) and the retrotransposons (class I) account for ∼20% and ∼40% of the insertions, respectively (Table S3). Using their chromosomal position, we localized each insertion within genes (within introns, UTRs, or exons) and intergenic regions. If more than 50% of an insertion length is outside the gene sequence, the insertion was recorded as intergenic insertion and if an insertion is within two overlapping genes, it was excluded. Lengths of TEs were calculated after excluding nested elements [5]. The canonical TE lengths were obtained from FlyBase (http://flybase.bio.indiana.edu/transposons/).

### Statistical analysis.

We analyzed the number of TE insertions with a GLM (function glm() in R, R Development Core Team, 2005) using a quasipoisson error distribution and log link. We used a backward procedure to refine models. We first included all predictor variables and their interactions. The main terms were entered in the order of decreasing deviance explained in separate analyses using only single terms (parsimony). We then removed those terms from a model that were not significant (unless they were main terms involved in a significant interaction term) and re-ran the model. We repeated this procedure until no more terms could be removed from a model. Significance of terms was tested with *F* tests.

## Supporting Information

Figure S1 Neighborhood Effect on Count of TE Insertion(s) The barplots represent the GLM coefficients for each genomic position around a focal gene or intergene (dashed line). The GLM (quasipoisson) includes the noncoding gene or intergene length, the recombination rate, the proportion of conserved elements, the chromosome (X versus autosomes), the tissue of expression (germline versus soma), and the tissue of expression of each of the 20 neighbor genes (indexation by their genomic positions from the focal gene). The figure illustrates the effect of gene expression of neighboring genes on TE number in a focal gene/intergenic region. For example, a focal gene has about 1.2 times more TE insertions when the right flanking neighbor is a germline-expressed gene. The stars indicate that GLM coefficients are significantly (*p* < 0.05) different from 0. The black bars are the positions that significantly explain variance in the GLM.
(71 KB PDF)

Figure S2 Short-Range Gene Clustering The figure shows the frequency with which focal germline- and soma-expressed genes (black and gray, respectively) in transcriptional and nontranscriptional territories are flanked by two genes with different types of expression (indicated on the *x*-axis). The horizontal bars indicate the expected frequencies under random distribution of genes in both territories. Significant departure from the expected frequencies are also depicted (Chi^2^: **, *p* < 0.01; *, *p* < 0.05; ., *p* < 0.1). Significant departures from the expectation occur only in transcriptional territories and show clustering by tissue of expression (e.g., germline-expressed genes have germline-expressed flanking neighbors).
(73 KB PDF)

Figure S3 Long-Range Gene Clustering The figure represents the frequency of germline- and soma-expressed genes (black and gray, respectively) at different positions neighboring a focal gene. Data are shown for focal germline- and soma-expressed genes (top and bottom row, respectively) within and outside transcriptional territories (left and right columns). The figure shows that within transcriptional territories, genes are organized in tissue-specific clusters: focal germline-expressed genes are surrounded by germline-expressed neighbors (and conversely for soma-expressed genes). In nontranscriptional territories, these clusters are virtually absent.
(90 KB PDF)

Figure S4 Noncoding Gene and Intergenic Region Length The figure represents the coefficients (±95% confidence intervals) of a log-gaussian GLM analysis of noncoding length of genes (introns + UTRs)/length of intergenic regions as a function of the factors “*tissue of expression*” (germline versus soma), “*element*” (gene versus intergenic), and “*territory*” (transcriptional territory versus nontranscriptional territory). Numbers below the labels on the *x*-axis indicate the number of intergenes and genes in each class, respectively. Significant terms in the GLM model include the double interaction element*territory (*F* = 57.5, *p* < 0.001), *tissue* *territory (*F* = 7.8, *p* < 0.01), tissue*element (*F* = 44.0, *p* < 0.001), and the three factors (*tissue*: F = 148.7, *p* < 0.001; *element*: F = 11.8, *p* < 0.001; *territory*: F = 22.3, *p* < 0.001).
(94 KB PDF)

Figure S5 Recombination Rate The figure represents the coefficients (±95% confidence intervals) of a gaussian GLM analysis of recombination rate as a function of the factors “*tissue of expression*” (germline versus soma), “*element*” (gene versus intergenic), and “*territory*” (transcriptional territory versus nontranscriptional territory). Numbers below the labels on the *x*-axis indicate the number of intergenes and genes in each class, respectively. Significant terms in the GLM model include the double interaction *tissue* *territory (*F* = 9.7, *p* < 0.01) and the factor *tissue* (*F* = 8.9, *p* < 0.01).
(94 KB PDF)

Figure S6 Sequence Conservation The figure represents the coefficients (±95% confidence intervals) of a quasibinomial GLM analysis of the proportion of conserved sequence as a function of the factors “*tissue of expression*” (germline versus soma), “*element*” (gene versus intergenic), and “*territory*” (transcriptional territory versus nontranscriptional territory). Numbers below the labels on the *x*-axis indicate the number of intergenes and genes in each class, respectively. All main effects in the model are significant (*tissue*, *p* < 0.01; *element*, *p* < 0.05; and *territory*, *p* < 0.01).
(93 KB PDF)

Table S1 Summary of the TE Insertions Included in Our Analysis by Element Family and Gene Expression (50 KB XLS)

Table S2 Number of Genes and Intergenic Regions with at least One TE and the Mean Number of Insertion per Gene or Intergenic Region (24 KB XLS)

Table S3 Numbers of TE Insertions by Element Class and Gene Expression (26 KB XLS)

Table S4 Properties of Transcriptional Territories In yellow, domains lacking either germline- or soma-expressed genes. In red, domains lacking both germline- and soma-expressed genes.
(63 KB XLS)

Table S5 Analysis of the Total Number of TE Insertions within Transcriptional Territories (GLM Family: Quasipoisson; Overdispersion = 4.6) (20 KB XLS)

Table S6 Analysis of TE Insertions with Transcriptional Territories (GLM Family: Quasipoisson; Overdispersion = 3.4) (17 KB XLS)

Table S7 Factors Affecting the Number of TE Insertions in Genes and Intergenic Regions: GLM Analysis with Levels of Expression in Germlines “Neighboring germline expression” is the sum of gene expression in germlines (ovary and testis) for the ten neighbors of a focal gene or intergenic region whereas “local germline expression” is the expression levels of a focal gene or the sum of expression levels of the two flanking genes of a focal intergenic regions. Coefficient signs of the GLM indicate the sign of linear coefficients associated with the terms of the model and hence indicate the direction (positive or negative) in which the different factors affect the number of TE insertions. Parentheses around the sign indicate that the linear coefficients were not significantly different from zero in the model.
(20 KB XLS)

Table S8 Factors Affecting the Number of TE Insertions in Genes and Intergenic Regions GLM analysis with levels of expression in ovary and testis, see Text S1 and Table S7 for details.
(21 KB XLS)

Table S9 Gene Clustering in Transcriptional and Nontranscriptional Territories Probability for a focal gene to have a least one germline- or soma-expressed gene between the two flanking neighbors.
(19 KB XLS)

Table S10 Factors Affecting the Total Number of TEs within Transcriptional Territories (GLM Analysis) The standard deviance of gene expression is an indirect measure of the degree of soma- and germline-expressed gene admixture in transcriptional territories. The sum of the gene expression in ovary and testis (germline expression) explains more variance of TE insertions within transcriptional territories than the separated effects (ovary expression and testis expression).
(19 KB XLS)

Text S1 Methods and Results of Alternative GLM Analyses This file contains descriptions of the methods and results of alternative GLM analyses, based on the level of expression of genes in the male and female germline (Tables S7–S10).
(37 KB DOC)

## Abbreviations

EST: expressed sequence tag
GLM: Generalized Linear Model
TE: transposable element
