# Supplementary figures and images for: Genome Organization and Gene Expression Shape the Transposable Element Distribution in the Drosophila melanogaster Euchromatin
Source: PLoS Genet. 2007 Nov 30;3(11):e210. doi: 10.1371/journal.pgen.0030210 (PMC2098804; doi:10.1371/journal.pgen.0030210)

Fold-change in TE number in a  
focal gene when presence  
of germline-expressed neighbor

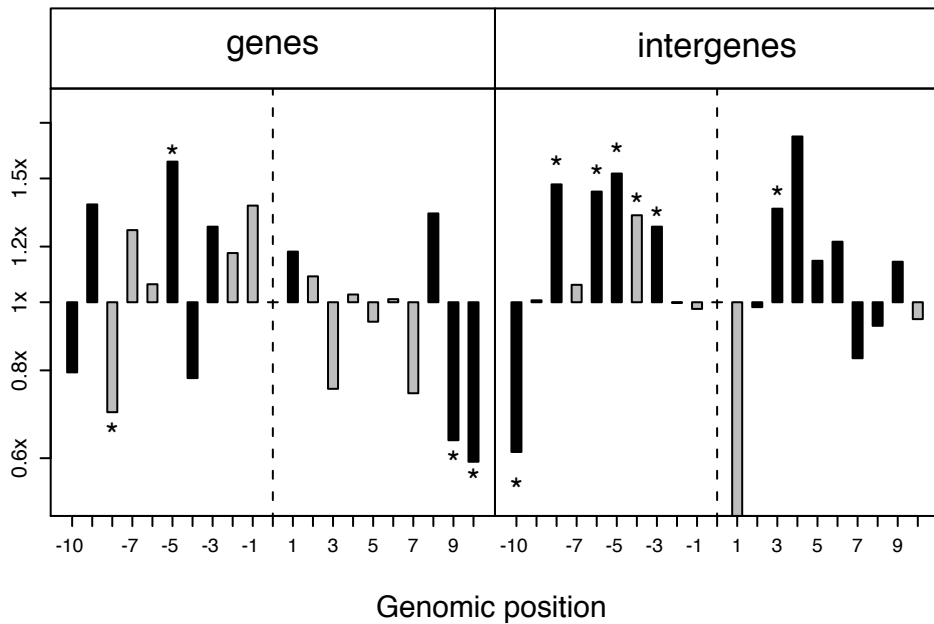

Supplement: Figure S1 — The barplots represent the GLM coefficients for each genomic position around a focal gene or intergene (dashed line). The GLM (quasipoisson) includes the noncoding gene or intergene length, the recombination rate, the proportion of conserved elements, the chromosome (X versus autosomes), the tissue of expression (germline versus soma), and the tissue of expression of each of the 20 neighbor genes (indexation by their genomic positions from the focal gene). The figure illustrates the effect of gene expression of neighboring genes on TE number in a focal gene/intergenic region. For example, a focal gene has about 1.2 times more TE insertions when the right flanking neighbor is a germline-expressed gene. The stars indicate that GLM coefficients are significantly (p < 0.05) different from 0. The black bars are the positions that significantly explain variance in the GLM. (71 KB PDF) [file pgen.0030210.sg001.pdf]

soma-expressed genes by class

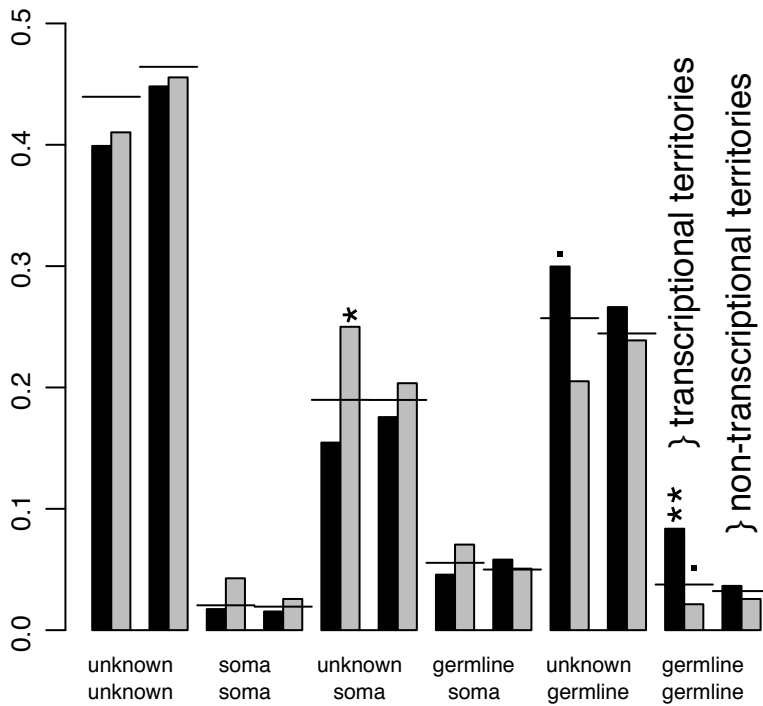

Expression of flanking genes

Supplement: Figure S2 — The figure shows the frequency with which focal germline- and soma-expressed genes (black and gray, respectively) in transcriptional and nontranscriptional territories are flanked by two genes with different types of expression (indicated on the x-axis). The horizontal bars indicate the expected frequencies under random distribution of genes in both territories. Significant departure from the expected frequencies are also depicted (Chi2: **, p < 0.01; *, p < 0.05; ., p < 0.1). Significant departures from the expectation occur only in transcriptional territories and show clustering by tissue of expression (e.g., germline-expressed genes have germline-expressed flanking neighbors). (73 KB PDF) [file pgen.0030210.sg002.pdf]

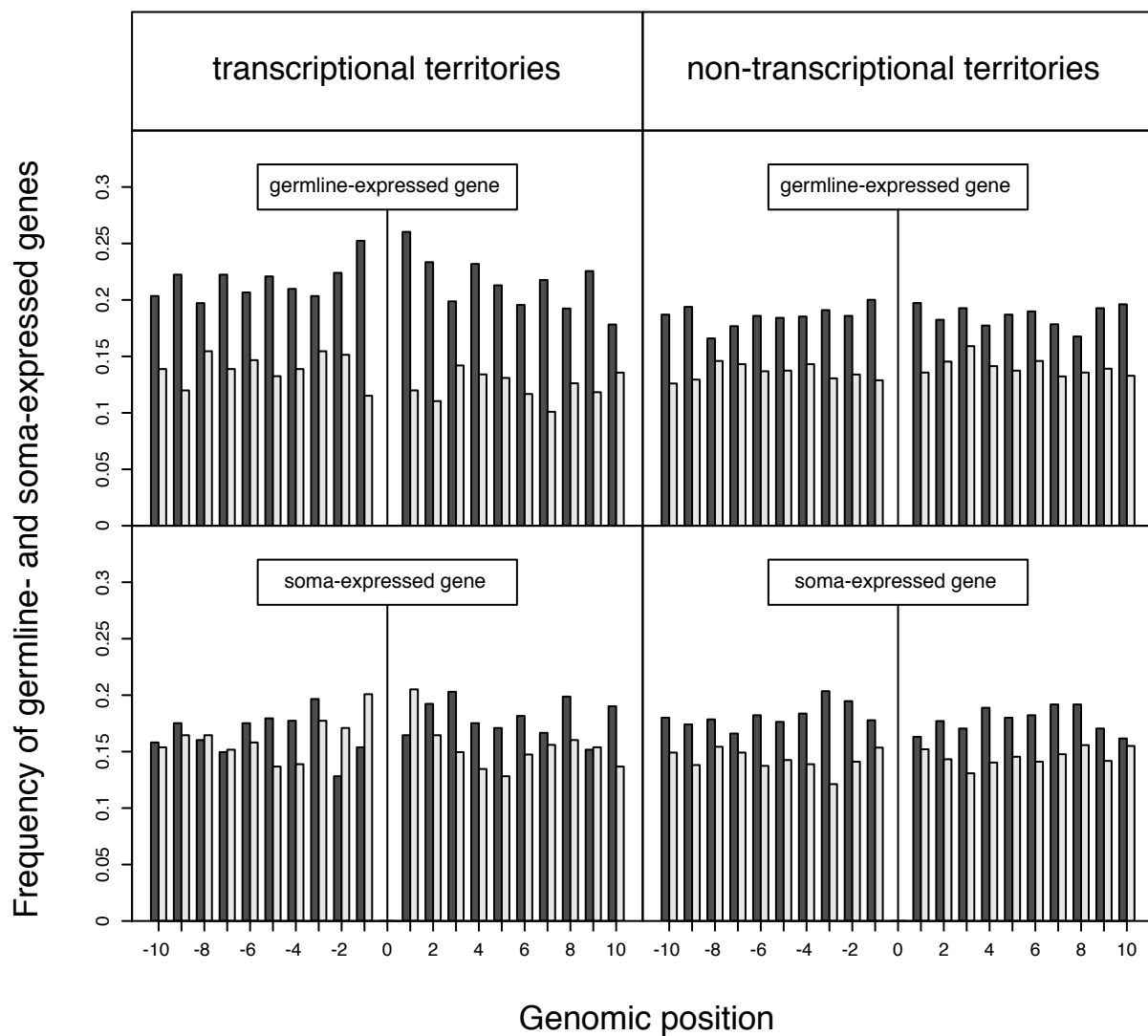

Supplement: Figure S3 — The figure represents the frequency of germline- and soma-expressed genes (black and gray, respectively) at different positions neighboring a focal gene. Data are shown for focal germline- and soma-expressed genes (top and bottom row, respectively) within and outside transcriptional territories (left and right columns). The figure shows that within transcriptional territories, genes are organized in tissue-specific clusters: focal germline-expressed genes are surrounded by germline-expressed neighbors (and conversely for soma-expressed genes). In nontranscriptional territories, these clusters are virtually absent. (90 KB PDF) [file pgen.0030210.sg003.pdf]

Length (bp)

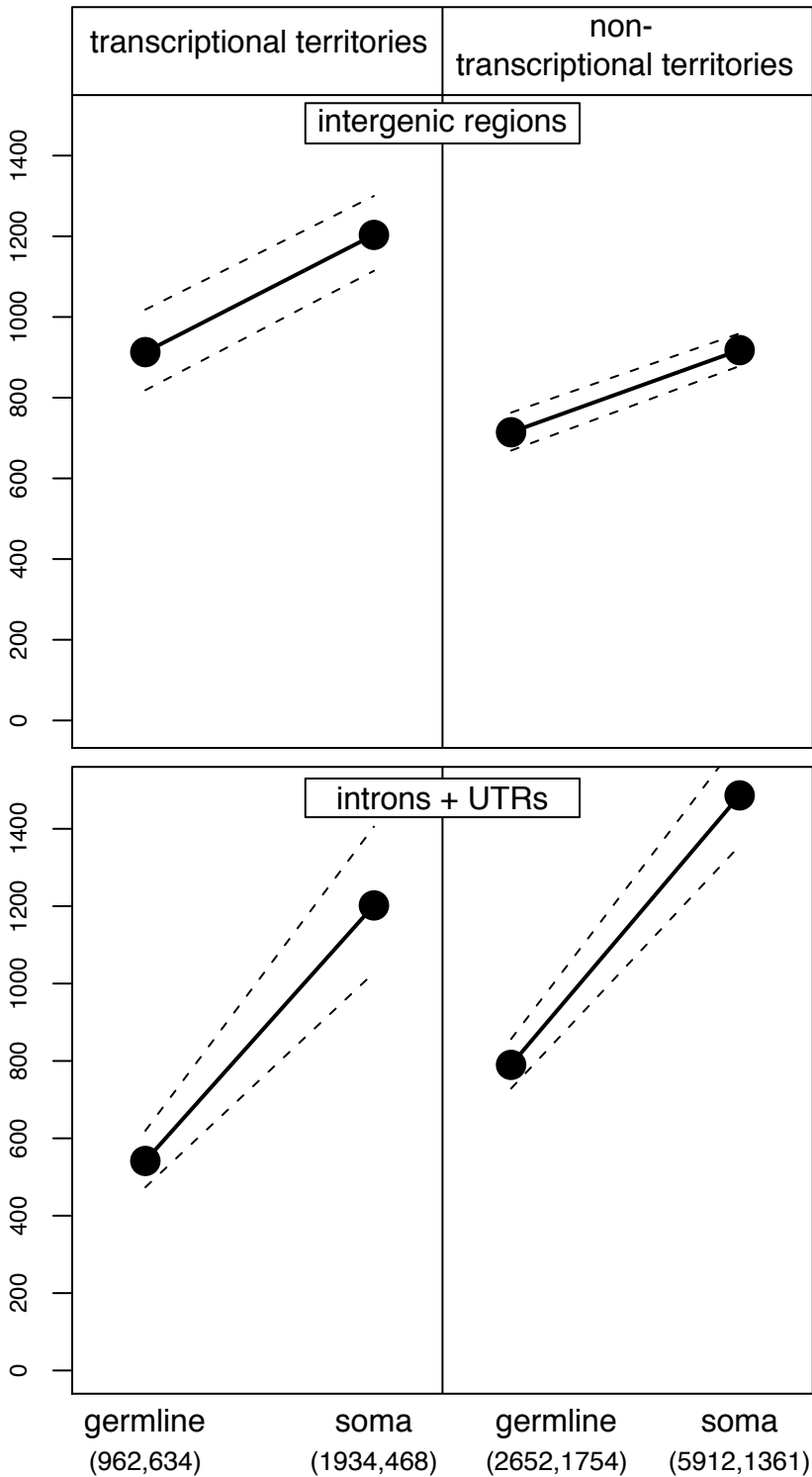

Supplement: Figure S4 — The figure represents the coefficients (±95% confidence intervals) of a log-gaussian GLM analysis of noncoding length of genes (introns + UTRs)/length of intergenic regions as a function of the factors “tissue of expression” (germline versus soma), “element” (gene versus intergenic), and “territory” (transcriptional territory versus nontranscriptional territory). Numbers below the labels on the x-axis indicate the number of intergenes and genes in each class, respectively. Significant terms in the GLM model include the double interaction element*territory (F = 57.5, p < 0.001), tissue *territory (F = 7.8, p < 0.01), tissue*element (F = 44.0, p < 0.001), and the three factors (tissue: F = 148.7, p < 0.001; element: F = 11.8, p < 0.001; territory: F = 22.3, p < 0.001). (94 KB PDF) [file pgen.0030210.sg004.pdf]

Recombination rate

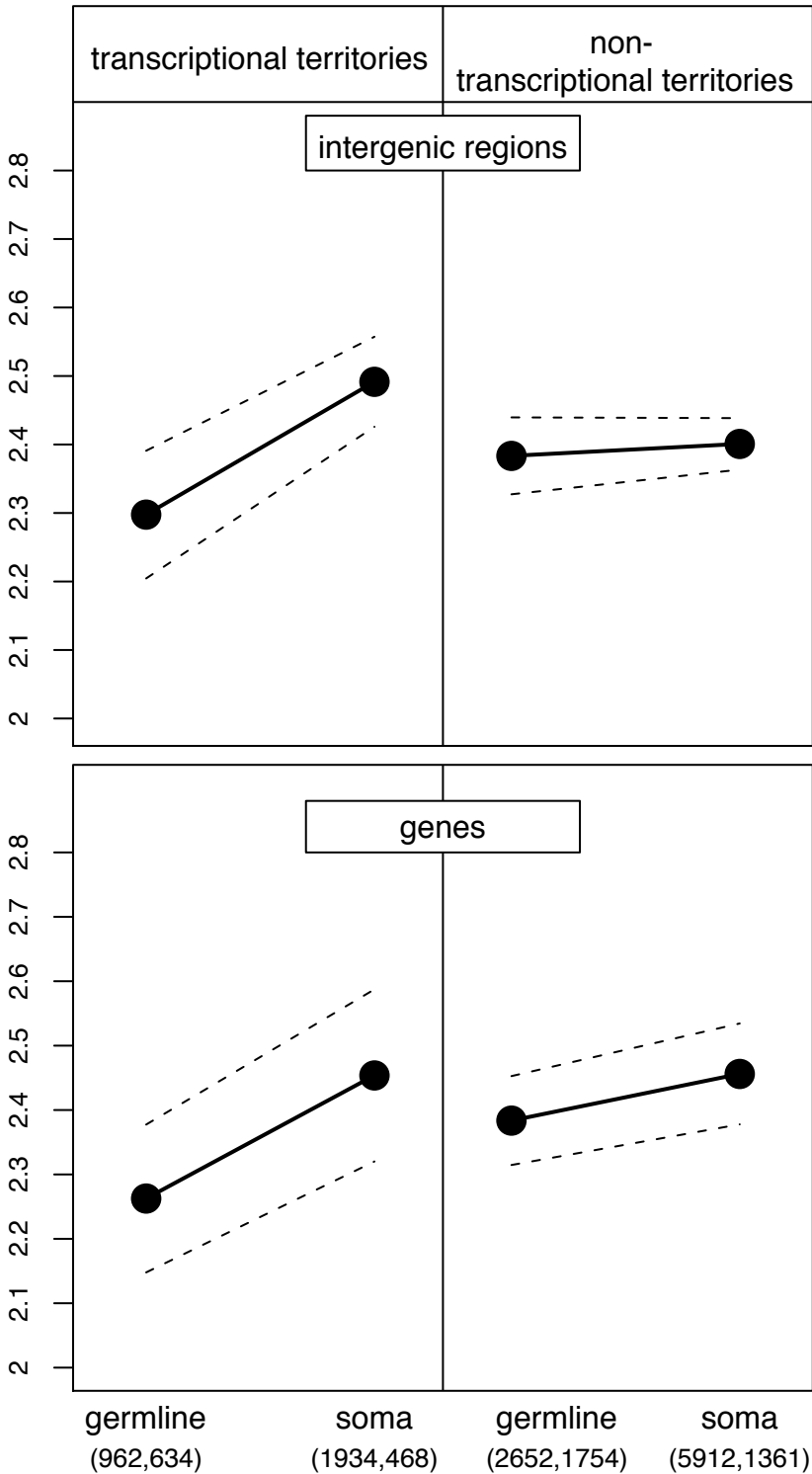

Supplement: Figure S5 — The figure represents the coefficients (±95% confidence intervals) of a gaussian GLM analysis of recombination rate as a function of the factors “tissue of expression” (germline versus soma), “element” (gene versus intergenic), and “territory” (transcriptional territory versus nontranscriptional territory). Numbers below the labels on the x-axis indicate the number of intergenes and genes in each class, respectively. Significant terms in the GLM model include the double interaction tissue *territory (F = 9.7, p < 0.01) and the factor tissue (F = 8.9, p < 0.01). (94 KB PDF) [file pgen.0030210.sg005.pdf]

Proportion of non-coding sequence conserved

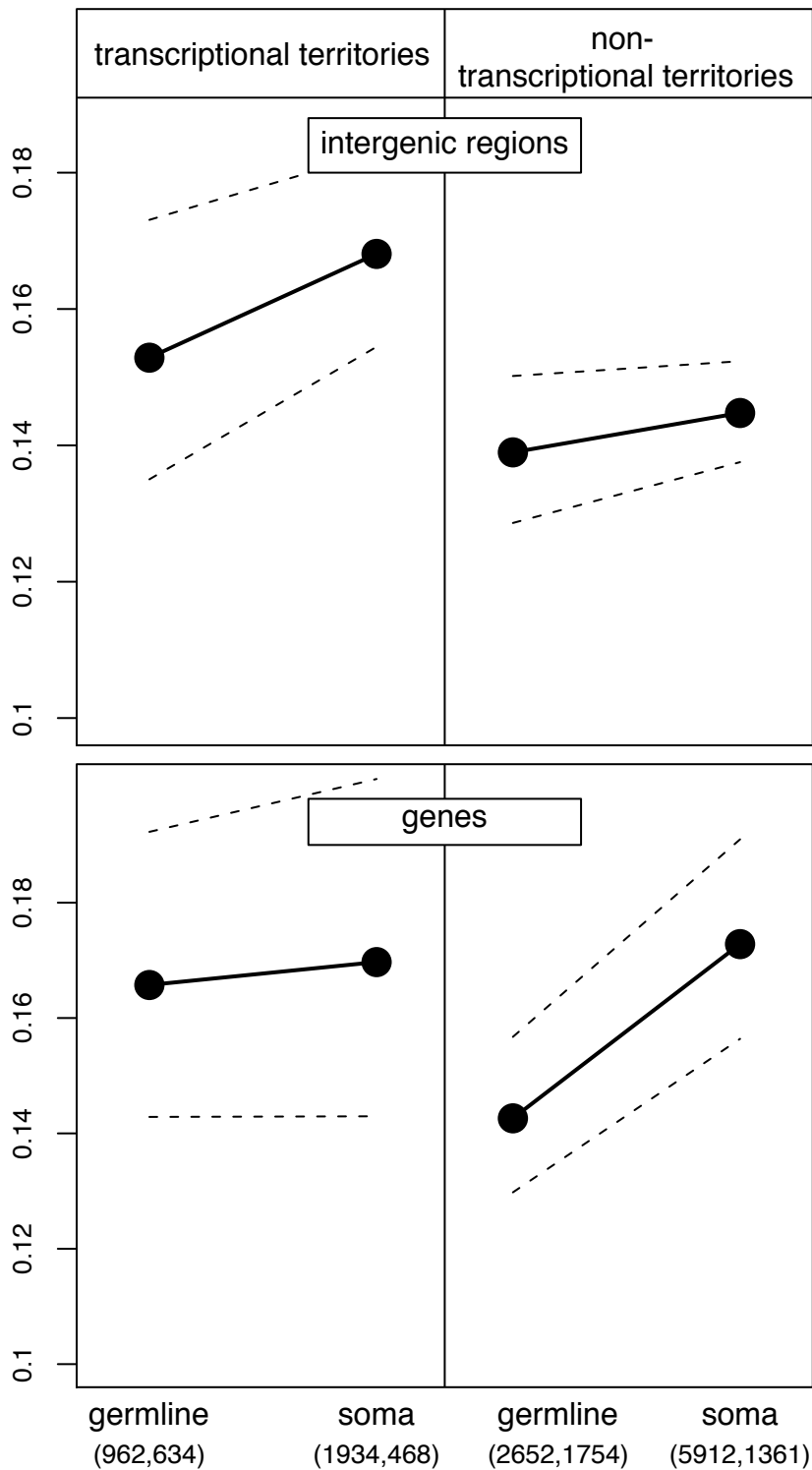

Supplement: Figure S6 — The figure represents the coefficients (±95% confidence intervals) of a quasibinomial GLM analysis of the proportion of conserved sequence as a function of the factors “tissue of expression” (germline versus soma), “element” (gene versus intergenic), and “territory” (transcriptional territory versus nontranscriptional territory). Numbers below the labels on the x-axis indicate the number of intergenes and genes in each class, respectively. All main effects in the model are significant (tissue, p < 0.01; element, p < 0.05; and territory, p < 0.01). (93 KB PDF) [file pgen.0030210.sg006.pdf]
