# Supplementary material for: Genome Organization and Gene Expression Shape the Transposable Element Distribution in the Drosophila melanogaster Euchromatin
Source: PLoS Genet. 2007 Nov 30;3(11):e210. doi: 10.1371/journal.pgen.0030210 (PMC2098804; doi:10.1371/journal.pgen.0030210)
Supplement: Text S1 — This file contains descriptions of the methods and results of alternative GLM analyses, based on the level of expression of genes in the male and female germline (Tables S7–S10). (37 KB DOC) [file pgen.0030210.sd001.doc]

**Text S1:**

**Reanalysis of TE distribution in the genome of *Drosophila melanogaster* using gene expression data (Affymetrix) instead of EST data**

***Purpose:***

The analysis presented in the main manuscript integrates gene expression as a variable defining genes as either germline- or soma-expressed. This classification was based on three EST libraries. In this document, we present an alternative analysis of TE density integrating gene expression as a continuous variable, representing the level at which genes are expressed in the germline. Expression measures were obtained from a recently published microarray dataset, containing the levels of gene expression in 10 different tissues (FlyAtlas, http://www.flyatlas.org/, Chintapalli *et al.* 2007). We performed this analysis in order to gauge whether our results were affected by the limitations of EST data. These limitations include a) the fact that two EST libraries were normalized, which prevents quantitative analysis of the level of gene expression, b) that only one somatic tissue (head) was considered, thereby increasing the risk of misattributing genes not expressed in the head to the germline tissue classes, and c) that EST libraries are biased towards highly expressed genes.

***Differences between EST and microarray approaches:***

The main difference concerns the definition of gene expression. In the EST-based analysis, we defined two distal classes of genes, those expressed exclusively in the germline and those expressed exclusively in the soma (a total of 4217 genes) that were expected to have contrasted effects on TE distribution. In the microarray analysis, we did not discard genes based on assumptions about their pattern of expression but based our analysis directly on the levels of gene expression in male and female germlines (testes and ovary). Accordingly, our dataset includes a much larger number of genes, totaling 13,046. For a detailed description of the approach, see the Methods section below.

The larger number of genes in this dataset, combined with the fact that microarray data are more fine-grained with respect to the number of tissues analyzed, also allowed us to perform analyses that separate the effects of male and female germline expression.

***Results:***

The results of the GLM analysis including general germline-gene expression are given in Table S7, those from the analysis separating expression levels in male and female germline (testes and ovary) are shown Table S8. Apart from the difference in the implementation of germline-gene expression, the variables included are identical to those in the main analysis (Table 1). Comparing Tables 1 and S7 shows that EST- and microarray-base approaches give very congruent results, despite the differences in the number of genes used and the definition of gene expression.

*Covariables*: The effects of recombination, non-coding length, proportion of conserved sequences and chromosomal location are virtually identical.

*Level of gene expression:* TE density increases in regions of the genome that are highly expressed in germlines. Just as in the main analysis (Table 1) the microarray-based analysis shows that TE density within and around a gene is affected mostly by germline expression of its neighbors rather than the level of germline-expression of the gene itself ('local germline expression').

*Ovary- versus testis-expressed genes*: As shown in Table S8, testis and ovary expression of genes increases the density of TEs in neighboring genes. However, this effect is stronger around ovary-expressed genes than testis-expressed genes. In addition, testis expression of a gene has a significant (negative) effect on the TE density with the gene itself, whereas ovary-expression shows no such effect (and accordingly dropped out of the analysis). This difference mirrors the difference in compactness of testis- and ovary-expressed genes (see below).

*Compactness and genome organization*: The analysis of microarray data confirms that local compactness varies with gene expression (Figure S7). The higher the expression level of a gene in somatic tissues, the longer are its non-coding sequence (introns + UTRs) and flanking intergenic regions. Furthermore, there are differences in compactness between testis- and ovary-expressed genes. Genes that are highly expressed in testes are very compact whereas genes highly expressed in ovaries have long non-coding sequences. The intergenic regions flanking highly germline-expressed genes are compact, irrespectively of whether expression occurs in the testes or in the ovaries.

The local compactness also depends on the amount of gene expression in the neighborhood (Figure S7) and thus, on genome organization. In transcriptional territories (see manuscript for details), germline-expressed genes have a high probability to be flanked by germline-expressed genes (and conversely for soma-expressed genes; Table S9). This genome organization, coupled to the compactness of germline-expressed genes and the level of germline expression, affects TE density within transcriptional territories. If each genes of a particular territory are highly expressed in germlines, its TE density is low because genes are compact. In the same way, if a particular territory contains only soma-expressed genes (no germline expression), its TE density is low because genes are not accessible in the germ cells. On the other hand, if a transcriptional territory contains genes with very heterogeneous levels of germline expression, indicating an admixture of germline- and soma-expressed genes, its TE density is high because the somatic genes are not compact and are under influence of germline neighbors (Table S10). In these transcriptional territories, the distinction between male and female germlines is less informative to explain TE density than their combined effects (levels of germline expression)

***Problems and limitations of the microarray analysis:***

While EST libraries suffer from the limitations outlined above, microarray data are associated with their own, different set of problems. Affymetrix GeneChips provide 'quasi-absolute' measure of expression levels within one sample. The levels are 'quasi-absolute' because although the relative contributions of each gene to the total expression in the sample are quite well inferred, these measures have no unit (for example, mRNA molecules/cell). It is therefore problematic to compare expression measures between samples and combine information from several Affymetrix arrays.

We combined the levels of ovary and germline expression (levels germline expression) by calculating the relative contributions of these two tissues to the expression in an adult whole-fly (see Methods). We thus obtained the total level of germline expression in a whole adult (expression by cells multiply by the number of cells in germline tissues). We are expecting that this measure is an indirect estimate of the accessibility of genes within the germ cells.

Finally, the relative effects of ovary and testis expressions on TE distribution (Table S8) might be affected by normalizations performed between chips.

***Methods:***

FlyAtlas (http://www.flyatlas.org/, Chintapalli *et al.* 2007) contains gene expression data covering ten distinct adult tissues (head, crop, midgut, Malpighian tubules, hindgut, thoracicoabdominal ganglions, thoracical carcass, testis, ovary, and male accessory glands). All tissues were dissected from 7-day-old Canton S adults. Except for the ovary, testis, and accessory glands, equal numbers of males and females contributed to each tissue sample. For each tissue, four independent biological replicates were analyzed on Affymetrix *Drosophila* Genome Arrays 2.0 (18,880 probe sets), reducing the effects of cross-contaminations.

*Level of expression within tissues*: We directly used the mean normalized values given by FlyAtlas as measures of gene expression. However, we considered the expression of a gene to be different from zero only if expression was detected in at least three out of the four biological replicates.

*Germline and somatic expression*: To combine the levels of expression of different tissues (i.e., testes and ovaries, or different somatic tissues) we needed to normalize expression data obtained from different samples in order for them to have directly comparable units (such as number of mRNAs per cell). When combining different samples from the same tissue, this can be achieved by standardizing the average or median expression level between samples. However, this is not a feasible approach when dealing with samples from different tissues, because organs differ in size (i.e. cell number) and can therefore not be assumed to contain identical amounts of mRNA. We therefore decided to normalize expression levels in each tissue by the contribution of the tissue to gene expression in a whole adult fly. More precisely, conversion-factors were obtained by regressing the expression level of tissue-specific genes in a single tissue sample on their expression level in a whole-fly sample. Ideally, such a regression would be linear (thus yielding a single conversion factor). However, saturation in the measurement of high expression levels means that a polynomial function was more appropriate. We performed one such regression analysis for each tissue and then used the polynomial function obtained to transform all expression measures obtained for that tissue.

*GLM analysis*: We constructed GLM models with quasi-poisson distributed error (function glm() in R). In refining a model, we used a backward procedure. We first included all predictor variables and their interactions. The main terms were entered in the order of decreasing deviance explained in separate analysis using only single terms (parcimony). We then removed those terms from the model that were not significant (unless they were main terms involved in a significant interaction term) and re-ran the model. We repeated this procedure until no more terms could be removed from the model.

***Literature:***

Chintapalli, V. R., Wang, J. and Dow, J. A. T. (2007). Using FlyAtlas to identify better Drosophila models of human disease. Nature Genetics 39: 715-720
